# That head lag is impressive! Infantile botulism in the NICU: a case report

**DOI:** 10.1186/s40748-023-00172-2

**Published:** 2024-01-02

**Authors:** Jaimie E Wardinger, Nada Darwish, Shaili Amatya

**Affiliations:** Division of Neonatal-Perinatal Medicine, Department of Pediatrics, Penn State Health Children’s Hospital, 500 University Dr, Hershey, PA 17033 USA

**Keywords:** Infantile botulism, BabyBIG, Infant, Sepsis

## Abstract

**Background:**

Infantile botulism (IB) is a devastating and potentially life-threatening neuromuscular disorder resulting from intestinal colonization by *Clostridium botulinum* and the resultant toxin production. It can present with constipation, descending paralysis, and, potentially, respiratory failure. Botulism is a diagnosis that is more commonly seen in the pediatric intensive care unit (PICU) or on the general pediatric wards and would not typically be managed in the neonatal intensive care unit (NICU), and therefore requires high clinical suspicion to ensure prompt diagnosis and treatment.

**Case presentation:**

We discuss a case where an infant from central Pennsylvania presented to a Level IV NICU rather than to the PICU for an evaluation for sepsis and was uniquely diagnosed with IB. The infant presented with poor oral feeding and reduced oral intake, hypothermia, and lethargy. His symptoms progressed into hypoxia and acute respiratory failure. Interestingly, this infant had no known exposure to honey or any other identifiable sources of botulism contact. The infant’s twin brother and the other infants who attended the mother’s in-home daycare remained asymptomatic. This infant was initially evaluated and managed for a potential infectious etiology. However, a diagnosis of IB was suspected, and was later confirmed through the detection of botulinum toxin in the infant’s stools. A high level of suspicion allowed for timely treatment with Botulism Immune Globulin neutralizing antibodies (BabyBIG), even prior to confirmatory testing. We describe the process of obtaining BabyBIG, as well as the natural course of illness after treatment in our patient who ultimately made a complete recovery.

**Conclusions:**

This case highlights the importance of considering infantile botulism as a diagnostic possibility even in the absence of risk factors, and the need for vigilance in diagnosing and treating this rare but potentially life-threatening condition. With timely recognition, subsequent treatment with BabyBIG, and supportive care, infants with infantile botulism can be expected to recover completely. This information is particularly important for neonatologists providing care for infants outside the neonatal period, especially during times of high patient census and resulting overflow of pediatric admissions in the NICU.

## Introduction

Infantile botulism (IB) is a rare neuromuscular disorder that occurs secondary to toxins produced by *Clostridium botulinum* [1]. IB typically affects children under 12 months of age and was first recognized in 1976 [2]. IB is the most common form of botulism in the United States, with approximately 70–100 cases occurring annually [2, 3], representing about 70% of reported botulism cases per year [3]. Most cases affect infants under 6 months of age, with a median age of presentation of 3–6 months [3, 4].

*C. botulinum* is an obligate, anaerobic, gram-positive bacillus that produces spores and is ubiquitous in the environment, such as soil and aquatic sediment [2–5]. *C. botulinum* has seven subtypes named A – G, but only subtypes A, B, E, and F are responsible for causing disease in humans [2–5]. Within the United States, subtypes A and B are responsible for IB, with most cases on the West Coast, specifically in California, occurring secondary to subtype A, and on the Northeast Coast, specifically in the Pennsylvania-New Jersey-Delaware area, occurring secondary to subtype B [2, 6]. Infants at the highest risk for contracting botulism seem to live in rural or farm environments, likely due to increased exposure to dust particles [2, 3]. Additionally, exposure to soil or dust from active construction sites also appears to increase the risk of contracting botulism [2, 3].

IB can be challenging to diagnose due to its initial vague presentation. It is often misdiagnosed as sepsis, meningitis, or encephalitis [2]. Symptoms can include any combination of constipation, poor feeding, a weak cry, lethargy, respiratory failure, and flaccid paralysis [2–9]. The generalized weakness typically starts with poor head control and progresses in a descending and symmetric fashion [5]. IB is commonly described as the ‘floppy baby’ syndrome in medical literature. However, botulism is not a diagnosis that would typically be on a neonatologists differential as it is a disease usually managed in the pediatric intensive care unit (PICU) or on the pediatric in-patient unit, and therefore has the potential to be misdiagnosed, or even missed if it were to present in the neonatal intensive care unit (NICU) [3]. Herein, we present an interesting case of an infant admitted to our NICU from an outside hospital to rule out sepsis and was found to have IB.

## Case Presentation

A 48-day-old male infant presented to the emergency room (ER) of an outside hospital (OSH) due to three days of lethargy, reduced oral intake, and reduced number of wet diapers. The initial evaluation in the ER included an abdominal ultrasound, which was unremarkable, and a respiratory viral panel (RVP), which was negative. The infant was sent home with recommendations for symptomatic management. Over the following day, the infant developed a progressively weaker suck and could only syringe-feed. He then had a choking episode with apnea and was brought back to the OSH ER. During this second visit, he was noted to be hypothermic to 32ºC rectally, hypoxic, and stridulous, with gurgling noises appreciated from his posterior oropharynx. The infant was admitted to the NICU for evaluation and management.

This patient was a twin born at 36 2/7 weeks gestation with an unremarkable pregnancy and delivery. He spent 9 days in the NICU for likely transient tachypnea of the newborn and required respiratory support via nasal cannula during that time. His twin brother was asymptomatic and well-appearing at home. The mother ran an in-house daycare located in central Pennsylvania. No other children attending the daycare were ill. The mother denied feeding her infant any honey. She denied any exposure to construction sites or soil but endorsed that they live near farmlands. Before the infant became ill, he was taking full bottles of either breast milk or formula and had regular bowel movements and normal tone. He was growing well and meeting his developmental milestones.

At the time of presentation, the infant had poor feeding and had not had a bowel movement in more than three days. He was afebrile and without rhinorrhea, cough, vomiting, or diarrhea. On physical examination, the infant was hypoxic to 70% with mild respiratory distress, and had bilateral ptosis, pooling of secretions in the oropharynx, generalized hypotonia with significant head lag, and minimal suck, gag, and Moro reflexes. He had 2 + patellar reflexes with weak hand and toe grasp reflexes.

The evaluation in the ER included a sepsis work-up with a blood and urine culture, which were negative, a urinalysis and a complete blood count, which were unremarkable, and a second RVP which was positive for rhino-entero virus (REV). A chest X-ray (CXR) was notable for a right upper lobe infiltrate suggestive of a pneumonia. Upon the infant’s presentation to the NICU, there were significant concerns for encephalitis, therefore, additional workup was done including a lumbar puncture for cerebrospinal fluid (CSF) culture, serum, and CSF PCR for herpes simplex virus (HSV), and skin surface cultures for HSV, all of which returned negative. A brain magnetic resonance image (MRI) was performed to evaluate for a stroke versus abscess as an etiology for the infant’s presentation and was normal.

Differential diagnoses included encephalitis or meningitis, which are pathologies typically seen in a NICU, unlike that of IB. However, due to the infant’s significant head lag and hypotonia, neuromuscular and metabolic disorders were also being considered including congenital myasthenia gravis, and spinal muscular atrophy. Although not typically seen in the NICU, botulism was also considered by the NICU fellow, a differential that was not agreed upon by majority of the NICU attendings at the time as it had never been diagnosed in this NICU previously.

The infant was placed on two liters per minute via nasal cannula for the hypoxia. He was warmed to achieve a core temperature of 36ºC rectally and was started on maintenance intravenous fluids. He was unable to tolerate oral feedings and could only be fed via a nasogastric tube (NGT).

The infant was treated with empiric antibiotics of Vancomycin, Ceftriaxone, and Acyclovir. There was continued right upper lobe opacification on his CXR, so he was continued on Ceftriaxone to manage a suspected superimposed bacterial pneumonia. Vancomycin and Acyclovir were discontinued after all other studies had returned negative.

The infant developed worsening respiratory distress, followed by apnea requiring emergent intubation and mechanical ventilation within less than 24 h from admission. His neurologic exam worsened, and the infant lost his gag and suck reflexes, and developed complete areflexia. At that point, the pediatric infectious disease team became involved and was convinced IB was the diagnosis based on the pattern of neurological exam changes, and the botulinum antitoxin (BabyBIG) was ordered from the Center for Disease Control (CDC) in California within 24 h of admission to the NICU. A stool sample was sent to the Department of Health in Pennsylvania for a confirmatory botulism toxin assay. As the infant had not stooled in over three days, a sterile water enema was performed to collect stool. Glycerin enemas are not recommended per the CDC because they tend to yield unsatisfactory samples. Instructions on how to properly obtain a stool sample for botulinum testing can be found on the website for Infant Botulism Treatment and Prevention Program – California Department of Public Health (https://infantbotulism.org/laboratorian/collection.php).

The infant further developed urinary retention requiring placement of a urinary Foley catheter. At that point, he demonstrated nearly complete paralysis with no spontaneous movements and very sluggish pupils that would become slower to respond with repeated exams, demonstrating neuromuscular fatigue.

The BabyBIG antitoxin arrived within less than 24 h of contacting the CDC. It was administered to the patient within less than 48 h from his admission to our NICU. Within three days of receiving BabyBIG antitoxin, our patient started to show clinical improvement with return of some spontaneous movements and tolerated weans on his ventilatory settings. He also tolerated initiation of enteral trophic feeds. The infant’s botulinum toxin assay returned positive for Botulism subtype B seven days after sending the test to the Department of health. On day nine from administration of BabyBIG, he was successfully transitioned to invasive-Neurally Adjusted Ventilatory Assist (NAVA) to allow monitoring of his diaphragmatic activity to predict extubation readiness. On day thirteen, he was successfully extubated to non-invasive NAVA and had three spontaneous bowel movements. Over the following two weeks, he was gradually weaned to room air and started working on oral feeds. Due to continued hypotonia, he received physical, occupational, and speech therapies. A barium swallow study demonstrated aspiration with oral feeding secondary to fatigue. He was therefore maintained on NGT feeds with gradual advancement of oral feeds. After 23 days of NICU stay, he was discharged home in room air, with a combination of oral and NGT feeds and outpatient follow-up appointments that included pediatric neurology, speech and occupational therapy, early intervention, and our NICU developmental clinic. Four months following his admission to our NICU, our patient is reported to be back to baseline without any residual comorbidities.

## Discussion

IB requires a high degree of suspicion to make the diagnosis and ensure prompt treatment without waiting for confirmatory testing [10]. IB can be a challenging diagnosis to make, as presenting symptoms are often vague. In this case report, an infant admitted to our NICU was diagnosed with and treated for IB, a diagnosis that is more commonly encountered in the PICU or on the general pediatric wards and typically would not be considered as a differential by most neonatologists as they are managing infants within the birth period onward, and usually are not managing infants who have been out in the community. However, during times of high pediatric census, such as respiratory season, NICUs may accept overflow admissions of pediatric patients and therefore should broaden their differential diagnoses when caring for infants outside of their trained speciality.

The presentation of IB may be confounded by non-specific symptoms such as lethargy and hypothermia [3], which can be suggestive of a serious bacterial infection, as was the case in our patient. Our initial diagnosis was encephalitis/meningitis, in addition to REV bronchiolitis and superimposed pneumonia. Therefore, an immediate botulism evaluation was not done. However, the worsening neurological symptoms occurring in the cephalic to caudal progression prompted a botulism workup within 24 h of admission. Retrospectively, it is likely that the pneumonia was secondary to aspiration caused by worsening tone and compromised gag reflex.

Following ingestion of *C. botulinum* spores, the spores germinate and then multiply in the intestinal tract [3–6, 8]. Due to the immaturity of the infant microbiome in conjunction with their developing immune systems and relative lack of gastric acidity, infants are thought to be particularly susceptible to intestinal colonization by *C. botulinum* [6]. Once the intestinal tract has been colonized, the botulinum neurotoxin travels to the bloodstream and lymphatic circulation and then to motor nerve endings [2–4, 8]. The neurotoxin irreversibly binds to cholinergic receptors in the presynaptic membranes of autonomic and voluntary neuromuscular junctions, which prevents the release of acetylcholine into the synaptic space, and ultimately leads to a descending flaccid paralysis [2–6, 8]. Compared to other neuromuscular disorders, the distinguishing feature of IB is symmetric bulbar palsy, which includes ophthalmoplegia, ptosis, fatigable pupillary response to light, decreased gag reflex, poor suck reflex, expressionless facies, and difficulty swallowing, all of which our patient eventually developed [5].

Botulism is one of the most lethal and potent poisons. A very low dose of 1 µg/kg can be lethal for an infant [3, 6]. The incubation period is thought to be between 3 and 30 days, with rare cases occurring soon after birth [2–4]. A well-known etiology of IB is ingestion of contaminated honey [6]. Generally,widespread educational campaigns have greatly reduced the consumption of honey in infants; however, certain ethnic communities are known to still use unpasteurized honey, placing these infants at increased risk for IB [9]. Unfortunately, a direct source for botulism is often never found. Our patient lives near farmlands in central Pennsylvania, which has a high incidence of IB, with the predominant subtype being type B, the same subtype our patient had [6]. Interestingly, our patient’s twin brother was unaffected, and no definitive source for this case of IB was ever identified.

Many infants with IB are brought to the ER following three or more days of constipation in the setting of decreasing oral intake and lethargy concerning for sepsis [1–4, 9, 11], which is also how our patient presented. Importantly, as this patient presented to an ER, he typically would not have been admitted to the NICU, as would be the case in the general community, and therefore an admission to a NICU creates a potential for missed diagnoses. He had a rapid respiratory decline secondary to diaphragmatic paralysis from the botulinum toxin necessitating mechanical ventilation, which is seen in approximately 50% of patients with IB [2–4]. Similar to what is reported in the literature, our patient had mostly negative blood work and imaging [1–4]. The positive RVP with REV is likely incidental.

Prior to 2003, the management of IB was purely supportive. However, the Food and Drug Administration (FDA) approved a new medication for the management of IB types A and B – a human-derived anti-botulism-toxin antibody known as intravenous botulism immunoglobulin (BIG-IV or BabyBIG) [2]. BabyBIG works by neutralizing free botulinum toxin so that no additional toxin can bind receptors at the neuromuscular junction, which then allows nerve terminals and motor end plates to regenerate [5, 7]. Only one dose of 50 mg/kg of BabyBIG is required, to be administered intravenously, as it has an approximate half-life of 28 days [10]. BabyBIG can be obtained by contacting the Infant Botulism Treatment and Prevention Program at any time. It is currently recommended that if IB is suspected, treatment with BabyBIG should be given promptly even before fecal samples are obtained or the diagnosis is confirmed, as delay in administration can greatly increase morbidity and mortality in affected neonates and infants [3, 4, 7]. This recommendation is largely based on the original study that demonstrated that early treatment with BabyBIG resulted in significant reduction in the length of hospital stay from 5.7 weeks to 2.6 weeks, along with a reduction in mechanical ventilation, duration of intensive care, tube feeding, and total charges of their hospital stays, when compared to delayed treatment [2].

IB has an excellent prognosis for those who receive BabyBIG, and the mortality rate in the United States for hospitalized infants is less than 1% [3, 4]. Per the California Department of Public Health, the average time from IB diagnosis to treatment with BabyBIG in the United States from 2013 to 2022 was 2.3 days [3], which matches closely with our patient who received BabyBIG within 48 h after admission. Recovery is a slow process, occurring over weeks to months, but typically infants make a complete recovery [2–4, 6, 10–12]. Infants will likely require physical and/or occupational and speech therapy services once discharged. However, they are expected to return to baseline unless severe complications occur [6].

## Conclusion

This case demonstrates the importance of having a high clinical suspicion for infantile botulism when a ‘floppy baby’ presents to your unit, as this allows for prompt diagnosis and treatment with overall improved outcomes. Infantile botulism is seen in infants typically under 12 months of age who present from the community when *C. botulinum* spores are ingested and can result in a rare and potentially life-threatening neuromuscular disorder. With timely recognition, subsequent treatment with BabyBIG, and supportive care, infants suffering from botulism can be expected to recover completely. This information is particularly important for neonatologists providing care for infants outside the neonatal period, especially during times of high pediatric census and resulting overflow of pediatric patient admissions in the NICU.

## Data Availability

A copy of consent form is available for review.

## List of abbreviations

IB: Infantile botulism
NICU: Neonatal intensive care unit
PICU: Pediatric intensive care unit
ER: Emergency room
OSH: Outside hospital
RVP: Respiratory viral panel
REV: Rhino-entero virus
CXR: Chest X-ray
CSF: Cerebrospinal fluid
HSV: Herpes simplex virus
NGT: Nasogastric tube
BabyBIG: Botulinum antitoxin
CDC: Center for Disease Control
NAVA: Neurally Adjusted Ventilatory Assist
FDA: Food and Drug Administration

## References

[1] Smith GE, Hinde F, Westmoreland D, Berry PR, Gilbert RJ (1989). Infantile botulism. Arch Dis Child.

[2] Rosow LK, Strober JB (2015). Infant botulism: review and clinical update. Pediatr Neurol.

[3] Horvat DE, Eye PG, Whitehead MT, Bharucha-Goebel D, Roth E, Anwar T (2023). Neonatal botulism: a Case Series Suggesting Varied presentations. Pediatr Neurol.

[4] Goldberg B, Danino D, Levinsky Y, Levy I, Straussberg R, Dabaja-Younis H (2023). Infant Botulism, Israel, 2007–2021. Emerg Infect Dis.

[5] Pifko E, Price A, Sterner S (2014). Infant botulism and indications for administration of botulism immune globulin. Pediatr Emerg Care.

[6] StatPearls. 2023.

[7] Rao AK, Sobel J, Chatham-Stephens K, Luquez C (2021). Clinical guidelines for diagnosis and treatment of Botulism, 2021. MMWR Recomm Rep.

[8] Fox CK, Keet CA, Strober JB (2005). Recent advances in infant botulism. Pediatr Neurol.

[9] Rossi M, Durrleman C, Hayat M, Roux CJ, Kossorotoff M, Gitiaux C (2022). Infant botulism: report of a misleading case and important key messages. Arch Pediatr.

[10] Gooding N, Kayani R, Whitehead L. Treatment of infantile botulism with botulism immune globulin (BabyBIG). 2018;104(7).

[11] Brown N, Desai S (2013). Infantile botulism: a case report and review. J Emerg Med.

[12] Ramroop S, Williams B, Vora S, Moshal K (2012). Infant botulism and botulism immune globulin in the UK: a case series of four infants. Arch Dis Child.

